# Particle Size Distribution of Plasma Lipoproteins in Donkeys from Death Valley Compared to a Sampling of Horses

**DOI:** 10.3390/ani12202746

**Published:** 2022-10-13

**Authors:** Erin L. Goodrich, Erica Behling-Kelly

**Affiliations:** Department of Population Medicine and Diagnostic Sciences, Cornell University College of Veterinary Medicine, Ithaca, NY 14850, USA

**Keywords:** donkey, equid, lipid, lipoprotein, metabolic syndrome, LDL, HDL, VLDL, liver, *Lipoprint* ^®^

## Abstract

**Simple Summary:**

Donkeys, like horses, belong to the Equidae family. Though they share some physical attributes, much of their physiology is quite different from horses. Both horses and donkeys can develop clinical issues associated with obesity; insulin dysregulation; and altered lipid metabolism, often termed equine metabolic syndrome or donkey metabolic syndrome, respectively. Unlike large breed horses, however, donkeys, ponies, and miniature horses are more susceptible to developing dyslipidemias. This study aimed to use a novel *Lipoprint*^®^ assay to compare the lipid profiles of apparently healthy donkeys and large-breed horses to establish a baseline for future work that may lead to the use of this test methodology for the evaluation and monitoring of equids with endocrine disorders.

**Abstract:**

The clinical evaluation of lipid metabolism in equids is often limited to the measurement of total cholesterol and triglyceride concentrations. This provides a limited picture of metabolic state and general health, given the continuous exchange of lipid species between various lipoproteins. Major lipoprotein classes in equids include high-density lipoprotein (HDL), intermediate-density lipoprotein (IDL), low-density lipoprotein (LDL), very low-density lipoprotein (VLDL), and chylomicrons (CM). Unlike large breed horses, donkeys are highly susceptible to hepatic lipidosis. Currently, serum triglyceride concentrations serve as a surrogate marker of hepatic lipid exportation. Both VLDL, indicative of hepatic exportation, and its metabolic end-product, LDL, are rich in triglycerides, and contribute to this value. Diagnostic assays that distinguish VLDL from LDL could be useful in better recognizing the hepatic pathology in donkeys. The compositional differences of lipoproteins across species limit the use of commercially available assays developed for the measurement of human lipoproteins in domestic animals. In this study, we evaluated a high-resolution polyacrylamide gel electrophoresis method (*Lipoprint*^®^) for separating major lipoprotein classes and sub-fractionating LDL and HDL based on particle size in a large group of donkeys, and compared the pattern to a representative set of horses. Donkeys proved an HDL-rich species, with HDL accounting for the bulk of all lipoproteins (average 78.45%, SD 6.6%, range 92.2–55%). VLDL accounted for a large portion of the total (average 21.6%, SD 6.6%, range 37.1–7.8%), with minimal amounts of LDL detected. The horses tested had higher proportions of VLDL as compared to donkeys (31.7% and 21.6%, respectively *p* = 0.00008). The later finding draws into question the purported relationship between VLDL, high triglycerides, and hepatic lipidosis, given the incidence of the disease in donkeys is far higher than in horses.

## 1. Introduction

Dyslipidemia refers to alterations in lipid distribution, namely triglycerides, cholesterol, and phospholipids [1]. Hyperlipidemia in equids is typically characterized by increased circulating triglyceride concentrations. The interpretation of hypertriglyceridemia must take into account the variability of reference ranges across diagnostic laboratories and across species within the Equidae family. Hyperlipidemia is a normal physiological response to negative energy balance. In certain situations, this response can become exaggerated and pathologic, potentially leading to grossly visible opalescent serum (hyperlipemia), the accumulation of fatty acids or triglycerides, and multiple-organ dysfunction [2,3]. Miniature horses, ponies, and donkeys are very prone to developing hyperlipidemia and hyperlipemia. These species often have some degree of insulin resistance, which further disables their ability to regulate lipolysis appropriately and compounds the issue [2,3]. Hyperlipemia can be a primary disease process or a result of a concurrent co-morbidity. In the absence of aggressive treatment, mortality rates of the disease in ponies, horses, and donkeys range from 43–80% [3,4,5]. Furthermore, there are many additional factors that can propagate the abnormal lipid metabolism in these cases. Physiologic stress (increased cortisol concentrations) and increased adrenocorticotrophic hormone (ACTH) in equids with pituitary pars intermedia dysfunction (PPID) can also exacerbate insulin resistance, precipitating the development of hyperlipidemia [3,5]. Donkey metabolic syndrome (DMS) and equine metabolic syndrome (EMS) provide clinical classifications for the complex association between obesity, insulin resistance, hyperinsulinemia, dyslipidemia, and endocrinopathic laminitis in these respective equids [1,4,5,6]. Given the insulin dysregulation that is common with DMS and EMS, these endocrinopathies also increase the risk for hyperlipidemia. Systemic inflammation, endotoxemia, and azotemia in equids and other species have also been shown to have effects on serum lipoprotein patterns, and are considered risk factors for hyperlipidemia [3,5,7,8].

Diagnosing hyperlipemia based on gross observation of serum is simple to do. However, lipemia is not always present in cases of hyperlipidemia or hypertriglyceridemia, and, thus, gross evaluation for lipemia remains an insensitive and inadequate method for the detection of hyperlipemia. Measuring triglyceride concentrations and, to a lesser extent, cholesterol concentrations can provide more useful information. The interpretation of these serum parameters is confounded by the variability that exists in reference ranges for both analytes across the various species of equids, breeds, and studies [9]. Both LDL and VLDL contribute to the overall serum triglyceride concentrations. Therefore, diagnostic assays that distinguish VLDL from LDL could serve as better means of identifying hepatic pathology associated with the increased export of triglycerides, as VLDL is more indicative of this process. In addition, the composition (often reflected in particle size) influences the biological activity of HDL, so assessing the particle size may serve as an important diagnostic tool.

Lipoprotein patterns can differ across different species, various breeds, and also between healthy and diseased states [10]. Thus, reagents developed for the quantification of human HDL and LDL are frequently proven inaccurate in veterinary species [11,12]. Several studies evaluating HDL and LDL in equids have not included or cited validation of the method for use in equids [13,14]. The *Lipoprint*^®^ assay uses high-resolution polyacrylamide gel electrophoresis to rapidly assess human LDL and HDL at the subfraction level. The method relies only on the physical characteristics of the lipoproteins, and uses no potentially species-specific reagents such as antibodies or precipitation methods. In this study, we evaluated this method for use in equids. We compared the electrophoretic particle size distribution between apparently healthy donkeys and large-breed horses. Defining these lipoprotein patterns to the subfraction level in apparently healthy equids will provide baseline information, so that future work may assess this assay as a diagnostic tool for the identification and/or prognostication of dyslipidemias in equids.

## 2. Materials and Methods

### 2.1. Animals

#### 2.1.1. Donkeys

Feral donkeys (*n* = 80) captured for removal from the Death Valley National Park range (Shoshone, CA, USA) during November and December 2018 were used for this study. They were co-mingled in pens with adult females and foals held together and separated from the adult male group. All donkeys were observed by veterinarians prior to sampling. The body condition score (BCS) on a 5-point scale [7,11], sex (*n* = 34 males, *n* = 46 females), and approximate age were recorded based on that veterinary evaluation. These samples were collected under the University of California Davis Institutional Animal Care and Use Committee protocol #20611.

#### 2.1.2. Horses

Healthy adult horses (*n* = 10) belonging to the Cornell University equine research herd in April 2021 were used for this study. The horses (*n* = 4 geldings, *n* = 6 females) comprised Warmbloods (4), Thoroughbreds (3), an American Paint (1), a Haflinger (1), and an Appaloosa/Hanoverian cross (1). All animals were observed by veterinarians prior to sampling, noted to appear healthy, and had no clinical signs of EMS or PPID. BCS was scored on a 9-point scale [15]. Horses were fed 1 cup of a commercial textured horse grain (Blue Seal Inspire Charger) with free-choice first-cutting grass hay daily (*n* = 6); or 1–4 quarts of a mixture of two commercial horse feeds (Blue Seal Inspire Trotter, a complete feed for horses) and Blue Seal Inspire Charger with free-choice first or second-cutting grass hay daily (*n* = 4)These samples were collected under the Cornell University Institutional Animal Care and Use Committee protocol 2007-0146 for diagnostic laboratory test validation and maintenance.

### 2.2. Blood Samples

A 10 mL blood sample was collected from each donkey and horse by venipuncture of the jugular vein and placed into EDTA tubes and red-top tubes (Beckton-Dickinson, Mississauga, ON, Canada), placed on ice, and shipped for overnight delivery to the Cornell University Animal Health Diagnostic Center (AHDC) within 24 h after collection (donkeys), or hand-delivered to AHDC within 4 h of collection (horses). Plasma and sera samples were separated by centrifugation (1500× *g*) for 20 min at 4 °C. The plasma and sera samples were aliquoted and stored at −80 °C until the time of analysis.

### 2.3. Laboratory Analyses

Triglyceride and cholesterol concentrations were assessed in all sera samples using the Cholesterol CHOD-PAP and Triglycerides GPO-PAP enzymatic colorimetric methods, respectively. In addition, the sera samples from the horses also underwent biochemical analysis, measuring the following parameters: sodium, potassium, chloride, bicarbonate, anion gap, sodium:potassium (Na/K) ratio, urea nitrogen, creatinine, calcium, phosphate, magnesium, total protein, albumin, globulin, albumin:globulin (A/G) ratio, glucose, aspartate aminotransferase (AST), sorbitol dehydrogenase (SDH), glutamate dehydrogenase (GLDH), gamma-glutamyl transferase (GGT), total bilirubin, direct bilirubin, indirect bilirubin, creatinine kinase (CK), iron, total iron-binding capacity (TIBC), and % iron saturation. All assays were performed on a Cobas 501 automated serum chemistry analyzer (Roche Diagnostics, Indianapolis, IN, USA). Chemical reagents were all Roche reagents, and the reactions are detailed in Table 1. All testing was performed by the Clinical Pathology Laboratory within the Animal Health Diagnostic Center (Ithaca, NY, USA) according to the laboratory’s standard operating procedures. The laboratory is AAVLD-accredited and participates in two external quality assurance programs.

### 2.4. Lipoprint^®^ Analysis

#### 2.4.1. LDL and HDL Subfraction Electrophoresis

The plasma samples were analyzed using the *Lipoprint^®^* LDL and HDL Subfraction Kits (cat 48-7002 and 48-9002, respectively) by the Quantimetrix Corporation (Redondo Beach, CA, USA), according to the manufacturer’s instructions. Each individual equid’s sample or aliquot (for stability and precision studies) was briefly thawed at room temperature. After the removal of the storage buffer, 25 μL of plasma was loaded onto the provided pre-cast linear polyacrylamide gels. Loading gel (200 µL for the LDL gel, 300 µL for the HDL gel) was then added to the top of the gel tube. The tubes were covered and inverted gently to mix the sample with the loading gel solution. The gels were photopolymerized using the provided lamp for 30 min. Samples were electrophoresed for 1 h at 3 milliamps per tube in the provided buffers (tris hydroxymethyl aminomethane and boric acid). Samples were run in random sets of 10 singletons along with a human plasma control sample provided by the manufacturer (LipoSep^®^, New Gloucester, ME, USA) for quality assurance purposes. Inter-assay CV was determined as part of an earlier study in dairy cattle [7].

#### 2.4.2. Densitometry

The tubes containing the electrophoresed and dyed lipoproteins were scanned in an ArtixScan M1 scanner that is provided with the *Lipoprint*^®^ system (Microtek International Inc., Hsinchu, Taiwan). The scanner is equipped with a cold-cathode fluorescent lamp. The scanned files were analyzed using the *Lipoprint*^®^ Software (LipowareTM, Redondo Beach, CA, USA) and the Image SXM program developed by Dr. Steve Barrett of the University of Liverpool (LDL Clinical: LW01-v.16-134). The intensity of each band was mathematically converted to a relative percentage value by the software. It should be noted that the software is automated for use with human samples and a known concentration of both LDL and HDL, which allows reporting of each subfraction in mg/dL. Routine biochemical tests to quantify human HDL and LDL do not transfer directly to bovine species. Therefore, a default value of 100 mg/dL was used as the total in the LDL and HDL electrophoretogram analyses. This allowed us to express each subfraction as a percentage of the total area under curve (AUC) as a raw value, rather than a converted concentration. If bands were too faint or run errors were detected by the software, band cut-offs were delineated using the manual measurement tool provided in the software package. This entailed visual inspection of the scanned gel image and corresponding software-generated plot to ensure that the demarcation of both the beginning and end of each individual peak aligned with the stained band on the gel image. In the LDL electrophoresis, VLDL, intermediate density lipoprotein (IDL), LDL subfractions 1–7, and total HDL were demarcated and assigned a numerical AUC value. The HDL band is the reference point for band enumeration in the LDL fractionation. In the HDL electrophoresis, subfractions 1–9 were demarcated and assigned a numerical AUC value. Albumin is the reference point for band enumeration in the HDL fractionation.

### 2.5. Data Analysis

Data were analyzed using Prism Graphpad software V9. (San Diego, CA, USA) Descriptive data (mean, SD, range) were generated for each fraction and subfraction. Normality was evaluated using the Shapiro–Wilk test. The individual lipoprotein fractions were compared between donkeys and horses using the Mann–Whitney test. A *p* < 0.05 was considered significant. Correlational analysis (Spearman rank) was performed to look for relationships between triglycerides and the beta lipoproteins.

## 3. Results

### 3.1. Study Subject Demographics

The approximate ages of the donkeys ranged from 4 months to 20 years, with a median of 6 years (*n* = 34 males, *n* = 46 females). Of the 54 donkeys for which the BCS data were approximated, the mean BCS was 3.06, with a median of 3.0 on a scale of 1–5 [16]. The actual ages of the horses ranged from 11 to 21 years, with both a mean and median of 16 years (*n* = 4 geldings, *n* = 6 females). The BCS of the horses ranged from 4 to 8, with a mean of 5.95 and median of 6 on a scale of 1–9 [15].

### 3.2. Laboratory Analyses

A total of 10 horses were originally enrolled in this study; however, one 17-year-old gelding Warmblood was excluded due to a GGT concentration above the established reference interval (RI) at the AHDC (88 U/L; RI 8–33 U/L), bringing our final horse population down to 9 total. The triglyceride and cholesterol concentrations of the horses largely fell within the established RIs at the AHDC, with only a single horse having a slightly low cholesterol concentration (67 mg/dL; RI 68–133 mg/dL). The remainder of the serum biochemistry panels on the horses also largely fell within normal RIs.

### 3.3. Donkey Lipoproteins

**Figure 1:**
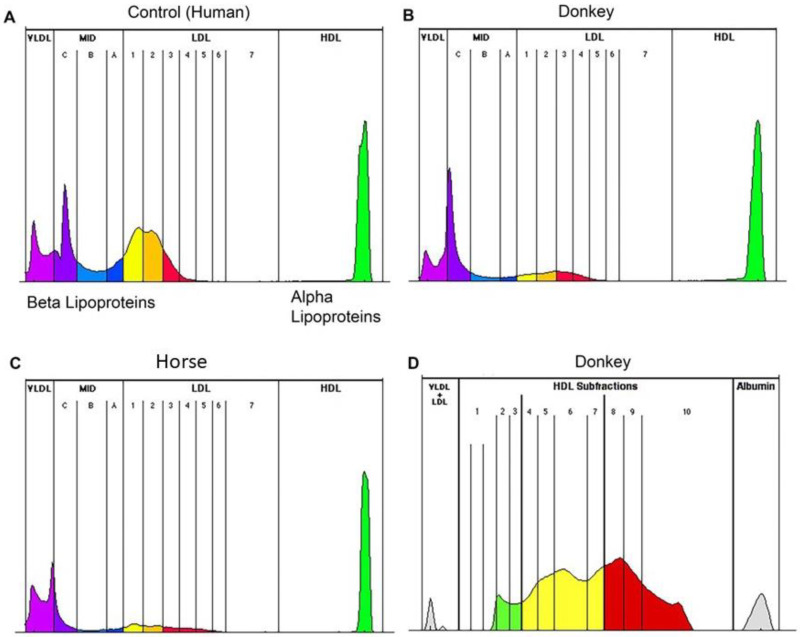
Representative lipoprotein plots from (**A**) human plasma control sample, (**B**) donkey, horse, (**C**) showing peaks for VLDL (light purple), midfractions (dark purple and light blue), LDL (yellow, orange, and red), and HDL (green), (**D**) depicting a representative HDL subfractionation from a donkey sample. HDL are divided into large particles (green), intermediated sized particles (yellow) and small sized HDL (red). Numbers indicate the subfraction.

**Figure 2:**
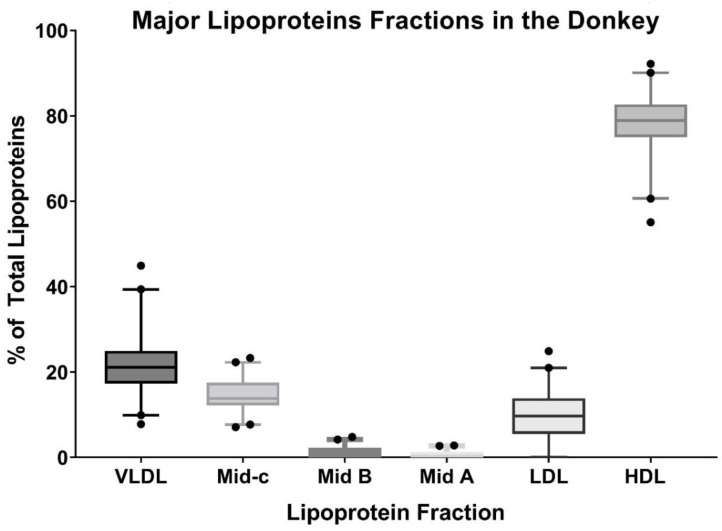
Summative data of the *Lipoprint*^®^ fractionation for all donkeys, displaying the median (bar) third and first quartiles (box), and 2.5–97.5% (whiskers) outliers are shown as individual points. The percent of total lipoproteins is generated from the area under the curves from the densitometry plots.

**Figure 3:**
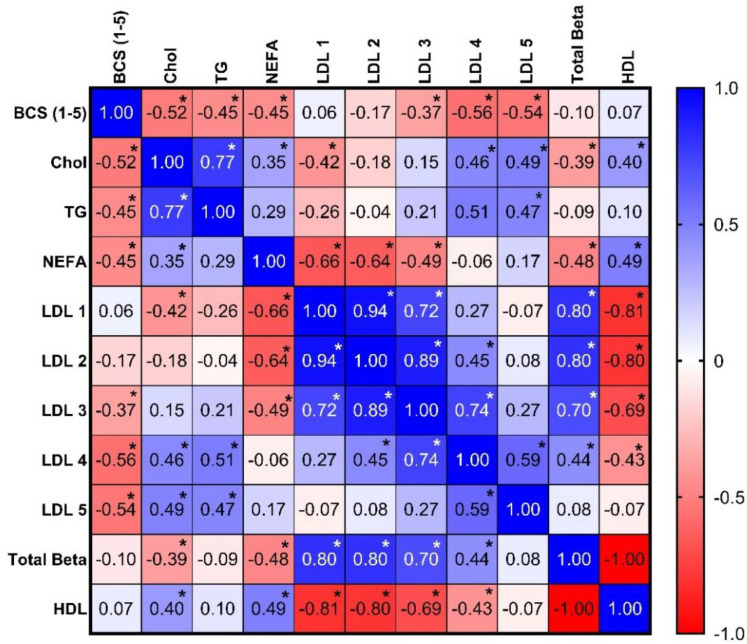
Correlation matrix comparing BCS, serum lipids, and lipoprotein classes/subfractions. Data are presented as the Spearman r. Asterisks indicate *p* < 0.05.

The *Lipoprint*^®^ method performed as expected and consistently separated donkey lipoproteins (Figure 1). HDL accounted for the majority of the lipoproteins (average 78.45%, SD 6.6%, range 92.2–55%). VLDL accounted for a large portion of the total as well (average 21.6%, SD 6.6%, range 37.1–7.8%), but with a higher spread of values across the population tested. Minimal amounts of LDL fractions were evident (Figure 2). In the HDL sub fractionation, donkeys had a wide distribution of particle sizes (1D). Serum TG, total cholesterol concentration, and beta lipoprotein fractions were all not normally distributed. No strong correlations between any fraction or subfraction and either triglyceride concentration or cholesterol concentration were found. Statistically significant, but weak-to-moderate, correlations were found between BCS and both triglycerides and cholesterol, as well as correlations between LDL 4 and 5. Total HDL had a strong negative correlation with LDL 1–3 fractions. Cholesterol and triglyceride concentrations shared a statistically significant, strong correlation (Figure 3). Body condition scores also did not correlate to triglyceride concentration nor VLDL.

### 3.4. Comparison to Equine Lipoproteins

**Figure 4:**
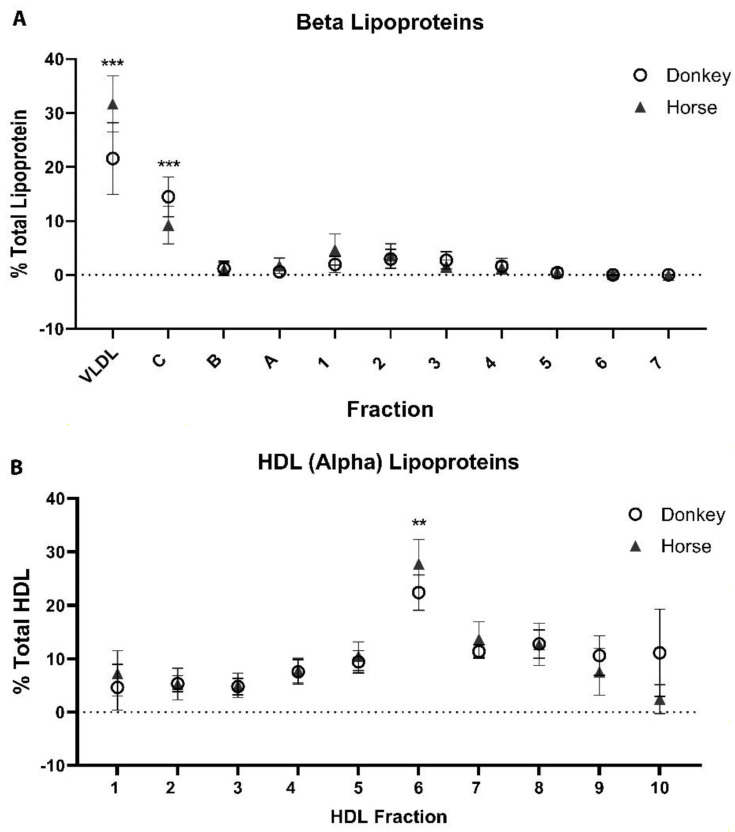
Summative data of LDL *Lipoprint*^®^ fractionation (**A**) and HDL *Lipoprint*^®^ fractionation (**B**). Data are depicted as the means and standard deviations. AUC, area under the curve. C, B, and A indicate the mid0fractions between VLDL and LDL. The remaining numbers are the numerical subfraction of the major lipoprotein class. ** *p* < 0.01, *** *p* < 0.001 (Mann–Whitney).

There were few differences between equine and donkey lipoprint subfractions using the *Lipoprint*^®^ method. In the LDL electrophoresis, the general size distribution of the largest beta lipoproteins was different in donkeys as compared to the horses. Donkeys had a higher proportion of beta lipoproteins in the Mid-C fraction, whereas the horses had a greater proportion in the VLDL fraction (Figure 4).

## 4. Discussion

Measuring triglyceride or cholesterol concentrations, and even total HDL and LDL, does not provide the most complete assessment of lipid metabolism. In fact, studies in dogs have shown that Miniature Schnauzers, a breed highly susceptible to hyperlipidemia, may have normal total cholesterol and triglyceride concentrations while also having important differences in lipoprotein patterns compared to other dog breeds [17]. Additionally, many investigations use reagents designed for the measurement of human lipoproteins without inclusion or reference to the validation of the method (refs). Many of the automated methods transfer poorly across species (refs). Evaluating lipid profiles in greater detail may be useful for species and breeds prone to dyslipidemias.

This study utilized a novel test method, the *Lipoprint*^®^ assay, to evaluate the relative quantification of the lipoprotein classes and subfractions present in equid plasma samples. Using this method, HDL represents the most abundant lipoprotein fraction for both the donkeys and horses in our study, which is consistent with the literature involving the lipoprotein patterns of horses and cows using other lipoprotein assays [18,19,20,21].

It is well established that donkeys, miniature horses, and ponies are predisposed to developing dyslipidemias, including hypertriglyceridemia [5]. A positive correlation has been reported between plasma HDL and triglyceride concentrations in Shetland ponies specifically [22]. The horses used in this study consisted of breeds that are not typically considered at high risk for EMS or hypertriglyceridemia, and they had no clinical signs of PPID [3,23,24]. Given the breeds in our study, and the fact that VLDL is rich in triglycerides and more indicative of hepatic lipid exportation than LDL, it is interesting that the horses had significantly more VLDL than the donkeys.

Human literature demonstrates that HDL functions as a scavenger of bacterial lipopolysaccharide (LPS), and has immunomodulatory effects, highlighting the possibilities that HDL may play in the treatment of sepsis and other inflammatory conditions [25]. Similar to canines, equids have HDL subfractions 8,9, and 10, which are absent in dairy cattle [7,26]. A recent canine study demonstrated a significant difference in HDL subfraction 9 between healthy dogs and dogs with septic abdomens, suggesting that furthering our understanding of lipid metabolism may assist in assessing illness severity in certain conditions, which could better inform the approach to therapy [26]. Future work could evaluate lipoprotein particle size distribution relative to inflammation biomarkers such as serum amyloid A (SAA) in equids to more accurately and timely identify and monitor the systemic inflammatory process.

Several limitations of this study should be mentioned. The sample size of horses and the breed distribution was limited, so any effects of age, gender, or breed on lipid profiles in horses cannot be determined in this study. Furthermore, the donkey population that we used in this study represented feral animals who had been living in desert conditions. The ages of the animals were only approximations, and it is unclear how well the results we obtained on these donkeys may translate to owned, domestic donkeys who receive different diets, levels of exercise, and healthcare. A previous study found that insulin dysregulation and dyslipidemia can all occur concurrently in horses, so another limitation of our study is the lack of exclusion of insulin dysregulation as a confounding factor, through the measurement of serum insulin and leptin concentrations in our enrolled equids [27]. The inclusion of body condition scoring for all equids enrolled would have also been beneficial, as lipid dysregulation is associated with obesity [19]. In addition, previous work using other lipid profiling methods have detected no significant differences in the LDL and HDL fractions in fed versus fasted horses, but the VLDL fractions were significantly higher in fasted horses when compared to fed [28]. The donkeys and horses in our study were sampled while forage was available, so we are unable to assess the effect of time since last feeding on the lipid profiles of these equids, and future work could take this into consideration.

Future *Lipoprint*^®^ studies comparing horses and donkeys with and without EMS or PPID are warranted. In addition, applying the assay to donkeys with and without clinical hyperlipemia would also further aid in our understanding of the potential for this assay as a useful diagnostic tool in that arena.

## 5. Conclusions

This study demonstrated the use of the *Lipoprint*^®^ assay as a useful and efficient tool for assessing lipoprotein profiles in donkeys and horses. The ability to assess LDL and HDL at the subfraction level, rapidly, is an improvement over previously available assays. Future studies assessing the application of this test in better-defined populations of horses and donkeys, both with and without EMS/DMS, PPID, obesity, inflammation, or hyperlipemia, are warranted.

## Figures and Tables

**Table 1:** Basic methodology for biochemical testing performed on the Roche Cobas.

Measurand	Method
Albumin	Bromocresol green dye-binding
AST	NADH oxidation
Electrolytes	Indirect ion-selective potentiometry
Bicarbonate	Phosphoenolpyruvate-carboxylase-based
Bilirubin, total	Jendrassik–Grof-based Diazo method (diazonium ion)
Bilirubin, direct	Jendrassik–Grof-based (diazotized sulfanilic acid)
Calcium	5-nitro-5′-methyl-BAPTA (NM-BAPTA)
Cholesterol	Cholesterol esterase
Creatinine kinase	Creatine phosphate cleavage
Creatinine	Modified Jaffe
GGT	L-gamma-glutamyl-3-carboxy-4- nitroanilde substrate
Glucose	Hexokinase
GLDH	Alpha-oxoglutarate substrate
Iron	Ferrozinc method
Magnesium	Xylidyl blue, diazonium salt
Phosphate	Ammonium molybdate
SDH	D-fructose substrate
TIBC	Unsaturated Fe binding
Total protein	Biuret
Triglycerides	Lipoprotein lipase
Urea nitrogen	Urease–kinetic

## Data Availability

The data presented in this study are openly available in FigShare at https://doi.org/10.6084/m9.figshare.21277716 (accessed on 9 October 2022).
